# The Main Coronary Artery (LMCA) Thrombus Presented as an ST Elevation Myocardial Infarction in a Hypercoagulable Patient

**DOI:** 10.1155/2012/579097

**Published:** 2012-12-24

**Authors:** Nithin Gottam, Douglas Stewart, Hiroshi Yamasaki, Mohamad Ajjour

**Affiliations:** Department of Cardiology, St. John Hospital & Medical Center, 22101 Moross Road, Detroit, MI 48236, USA

## Abstract

Left main coronary artery (LMCA) thrombus is a clinically rare event and is thought to be secondary to plaque rupture with subsequent thrombus formation, persistent hypercoagulable state, cocaine use, or vasospasm. (Klein et al., 2008) here we present a case of LMCA thrombus that presented as an ST elevation myocardial infarction (STEMI) in a hypercoagulable patient.

## 1. Introduction

Left main coronary artery thrombus is a clinically rare event. Clinical presentation often occurs with ST elevation myocardial infarction (STEMI), non-ST elevation myocardial infarction (NSTEMI), unstable angina (USA), cardiogenic shock, or sudden cardiac death [1]. The incidence is estimated to be ~0.8%, but this is thought to be an underrepresentation given sudden LMCA thrombus may present with sudden cardiac death [1]. Pathophysiology of event is thought to be secondary to plaque rupture with subsequent thrombus formation, persistent hypercoagulable state, cocaine use, or vasospasm [1]. Here we present a case of LMCA thrombus in the setting in a patient with known hypercoagulable state that presented as STEMI.

## 2. Case Report

A 50-year-old African American female, who is a Jehovah's witness, with past medical history of systemic lupus erythematosus (SLE), hypertension, hyperlipidemia, bilateral pulmonary embolism in 2011, erosive gastritis (diagnosed in 1/2012), and crescentic glomerulonephritis presented to the emergency room (ER) with complaints of 1-hour chest pain that started with exertion. The chest pain was described as pressure-like with radiation down the left arm, which started with walking 1 block. Patient describes pain, also associated with shortness of breath and diaphoresis, as resolving with rest.

On initial examination, vital signs were 112/76 mmHg, pulse of 82 beats/min, respiratory rate of 13 breaths/min, and temperature was 98.7 F. Pt was in moderate distress. Cardiac auscultation demonstrated no murmur, rub, or gallop. 12-lead electrocardiogram (EKG) demonstrated ST elevation in inferior leads and ST depression in lateral leads. Given her chest pain and EKG, she was urgently taken to cardiac catheterization for emergent coronary angiography. Initial cardiac Troponin-T level was 8.93 ng/L (normal <0.05).

**Figure 1:**
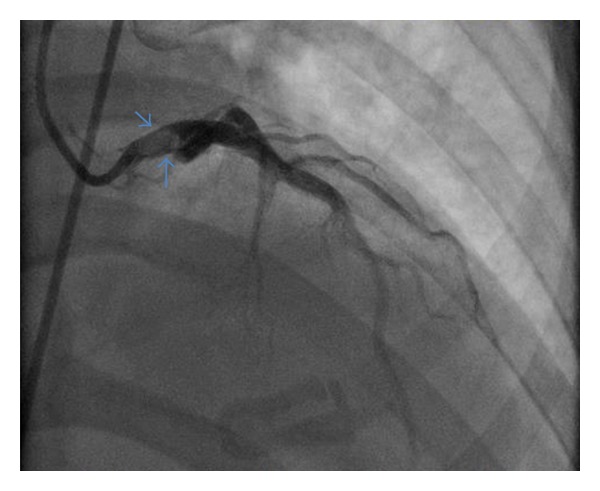
Angiogram in RAO & CRANIAL view demonstrating large proximal thrombus attached to the superior and proximal walls of the left main coronary artery, as indicated by arrows.

The coronary angiogram demonstrated a large proximal thrombus attached to the superior and anterior wall of the LMCA, with distal TIMI III flow (Figure 1). The remaining coronary arteries were found to have mild luminal irregularities. Thrombectomy ×3 was attempted, with minimal improvement in size of thrombus. Intravascular ultrasound (IVUS) was used to confirm the large LMCA thrombus. In addition, IVUS did not demonstrate endothelial damage proximal or distal, to the thrombus. Given her history of erosive gastritis and refusal of blood products, the decision was made not to treat her with intracoronary tissue plasminogen activator (tPA). Given clinical status, decision was made to transfer her to tertiary medical center on Heparin drip, Eptifibatide drip, and Aspirin for further care.

Patient was brought to the cardiovascular intensive care unit where her vital signs were 108/75 mmHg, pulse of 81 beats/min, respiratory rate of 16 breaths/min, and temperature was 98.2 F. The remainder of the physical exam was unchanged. Initial EKG demonstrated resolution of both ST elevations in inferior leads and ST depression in lateral leads. Patient was started on a nitroglycerin gtt, after which she reports resolution of chest pain. A bedside two-dimensional echocardiogram was performed, which demonstrated left ventricular ejection fraction of 55%, apical inferoseptal, and apical inferior akinesis. Cardiovascular surgery was consulted for possible coronary arterial bypass grafting surgery (CABG). However, given the situation that the chest pain had resolved, the patient's status of Jehovah's Witness, history of bleeding while on anticoagulation, and given TIMI III flow distal to the thrombus would lead to premature graft occlusion, the decision was made to recommend conservative management. Given her history of chronic bilateral pulmonary embolism, SLE, and crescentic glomerulonephritis, a hypercoagulable workup was performed and demonstrated positive anticardiolipin IgG & IgM. After 3 days of treatment with heparin, she was taken back to the cardiac catheterization laboratory for repeat angiogram which demonstrated continued LMCA thrombus attached to superior/anterior wall of proximal LMCA and TIMI III flow distal to thrombus, with mild luminal irregularities of the remaining coronary arteries. Intracoronary tPA was deferred due to concern of bleeding in setting of Jehovah's Witness. Given her hypercoagulable state in setting of LMCA thrombus, she was started on Warfarin. After extensive discussion with patient, family, and hematology specialist, decision was made to continue life-long anticoagulation. Patient was discharged home in stable condition on aspirin and warfarin.

## 3. Discussion

Left main coronary artery thrombus is caused by a variety of pathologic causes including secondary to plaque rupture with subsequent thrombus formation, persistent hypercoagulable state, cocaine use, and vasospasm [1]. The incidence of LMCA thrombus is unknown, but is thought to be ~0.8% [1]. This is likely an underrepresentation given that presentation may be sudden cardiac death that may not be classified as being secondary to LMCA thrombus [1]. Clinical presentation varies and often includes STEMI, NSTEMI, USA, and sudden cardiac death. Vasospasm reduces lumen diameter and blood flow leading to increased cause of thrombus formation [2]. 

Approach to management of LMCA thrombus is often conservative in patients with following conditions: no evidence of ongoing ischemia, absence of significant flow-limiting disease in LMCA, absence of significant atherosclerotic disease in remaining portions of coronary tree, and TIMI III flow in LAD and LCx arteries [1].

Conservative management includes 24–48 hrs of IV heparin, glycoprotein IIb/IIIa inhibition, and aspirin [3]. After conservative treatment, patients should undergo repeat angiography to reevaluate the thrombus to determine resolution versus further intervention [1]. If resolution has occurred, Clopidogrel is recommended to be added for further antiplatelet treatment. With regards to antiplatelet treatments, Abciximab is recommended given it can be reversed with platelet transfusions if emergent CABG is necessary [1]. IVUS use is controversial, but its use provides accurate assessment of plaque burden and excludes LMCA dissection, and resolution of thrombus postmedical treatment [1]. 

Non-conservative management includes percutaneous intervention including stenting, and mechanical aspiration thrombectomy [1, 2]. In addition, pharmacological treatment with intracoronary streptokinase and tPA delivery. Surgical intervention includes emergent CABG.

The presentation of our patient demonstrated an acute left main coronary artery thrombus in the setting of an acute myocardial infarction in a hypercoagulable patient. We present this case to discuss the difficulty of treating LMCA thrombus in a hypercoagulable patient, with the additional complexity of the patient being a Jehovah's Witness.
